# Myoelectric interface training enables targeted reduction in abnormal muscle co-activation

**DOI:** 10.1186/s12984-022-01045-z

**Published:** 2022-07-01

**Authors:** Gang Seo, Ameen Kishta, Emily Mugler, Marc W. Slutzky, Jinsook Roh

**Affiliations:** 1 Department of Biomedical Engineering, Cullen College of Engineering, University of Houston, 3517 Cullen Blvd, SERC Room 2011, Houston, TX 77204-5060 USA; 2 Department of Neurology, Northwestern University, 320 E. Superior Ave., Searle 11-473, Chicago, IL 60611 USA; 3 Department of Neuroscience, Northwestern University, Chicago, IL USA; 4 Department of Physical Medicine and Rehabilitation, Northwestern University, Chicago, IL USA; 5 Department of Biomedical Engineering, Northwestern University, Evanston, IL USA

**Keywords:** Muscle synergy, Intermuscular coordination, Myoelectric computer interface, Stroke motor rehabilitation

## Abstract

**Background:**

Abnormal patterns of muscle co-activation contribute to impaired movement after stroke. Previously, we developed a myoelectric computer interface (MyoCI) training paradigm to improve stroke-induced arm motor impairment by reducing the abnormal co-activation of arm muscle pairs. However, it is unclear to what extent the paradigm induced changes in the overall intermuscular coordination in the arm, as opposed to changing just the muscles trained with the MyoCI. This study examined the intermuscular coordination patterns of thirty-two stroke survivors who participated in 6 weeks of MyoCI training.

**Methods:**

We used non-negative matrix factorization to identify the arm muscle synergies (coordinated patterns of muscle activity) during a reaching task before and after the training. We examined the extent to which synergies changed as the training reduced motor impairment. In addition, we introduced a new synergy analysis metric, disparity index (DI), to capture the changes in the individual muscle weights within a synergy.

**Results:**

There was no consistent pattern of change in the number of synergies across the subjects after the training. The composition of muscle synergies, calculated using a traditional synergy similarity metric, also did not change after the training. However, the disparity of muscle weights within synergies increased after the training in the participants who responded to MyoCI training—that is, the specific muscles that the MyoCI was targeting became less correlated within a synergy. This trend was not observed in participants who did not respond to the training.

**Conclusions:**

These findings suggest that MyoCI training reduced arm impairment by decoupling only the muscles trained while leaving other muscles relatively unaffected. This suggests that, even after injury, the nervous system is capable of motor learning on a highly fractionated level. It also suggests that MyoCI training can do what it was designed to do—enable stroke survivors to reduce abnormal co-activation in targeted muscles.

*Trial registration* This study was registered at ClinicalTrials.gov (NCT03579992, Registered 09 July 2018—Retrospectively registered, https://clinicaltrials.gov/ct2/show/NCT03579992?term=NCT03579992&draw=2&rank=1)

## Background

Stroke, the largest cause of long-term disability worldwide, often damages motor pathways in the brain and induces abnormal spatiotemporal patterns of co-activation across arm muscles [1], also called abnormal muscle synergies [2–5]. Using dimensionality reduction methods, such as non-negative matrix factorization (NMF) or principal component analysis (PCA), several studies characterized the stroke-induced abnormal muscle synergies based on the electromyography (EMG) signals of the arm muscles [6–8]. Commonly observed abnormal muscle synergies after stroke include coupling of activity between elbow flexors and shoulder abductors during isometric torque generation [1, 4] and dynamic reaching [9]. Moreover, stroke induces co-activation of the anterior, middle, and posterior deltoid during the stable force maintaining phase of the isometric reaching task [7]. This co-activation inhibits the ability to flex the shoulder maximally. These abnormal patterns contribute to motor impairment and reduced function after stroke, partially by limiting range of motion during reaching [10, 11]. Thus, targeting these abnormal patterns is a potential new avenue for stroke rehabilitation.

Our previous study developed a myoelectric computer interface (MyoCI) paradigm to reduce abnormal co-activation and thereby impairment [12, 13]. Mugler et al. [13] examined 6 weeks of in-lab MyoCI training in thirty-two chronic stroke survivors with moderate-to-severe impairment. Before the training, each participant went through a screening process to identify the three most abnormally co-activating arm muscle pairs (defined by pairwise correlation coefficients) during free-reaching to targets, placed at waist and shoulder height, in front of and lateral to the impaired limb. After the screening, the participants were randomized to three different training groups (60 and 90 min using isometric activation; 90 min with activation during unrestricted movement). The participants were trained on and learned to activate each identified muscle pair separately for 2 weeks. At the end of the MyoCI training, participants had reduced arm impairment (measured using the Fugl-Meyer Assessment), improved motor function and elbow range-of-motion, and reduced spasticity (measured with the Modified Ashworth Scale). However, it remains unclear how MyoCI training affected intermuscular coordination in the arm in reaching movements.

Here, we investigate to what extent MyoCI training changed intermuscular coordination. We assessed the composition and number of muscle synergies as effects on the more global arm muscle network. Further, we augmented our muscle synergy analysis by developing the disparity index (DI), which measures the disparity between the synergy activation weights of each pair of muscles trained.

## Methods

### Participants

Thirty-three stroke survivors (15 women; age ranging from 27 to 75 years; mean of 6.5 years since stroke) participated in the original experiment [13]. The time since stroke was 3.8 ± 6.2, 4.3 ± 4.1, and 5.3 ± 3.2 years for 60I, 90I, and 90 M groups, respectively (mean ± SEM) and ranged from 11 to 314 months. All participants completed the entire 6 weeks of training; 32 completed the 10-week evaluation. An occupational therapist performed the Fugl-Meyer Assessment of the upper extremity (FMA-UE) on the impaired arm to assess the motor impairment of participants. We included adult, chronic stroke survivors (at least 6 months from stroke onset) who had persistent moderate to severe arm impairment (FMA-UE, 8-40/66) and increased arm tone. Full inclusion/exclusion criteria can be found in Mugler et al. [13]. The trial is registered at ClinicalTrials.gov, NCT03579992. The study protocol was approved by the Northwestern University Institutional Review Board, and each participant gave written informed consent prior to eligibility assessment.

### Training and assessment paradigm

The MyoCI training involved using EMG envelopes of the targeted muscle pair to move a cursor to different targets in a custom-built game to reduce abnormal co-activation. The cursor started in the bottom left of the screen when the muscles were at rest. EMG amplitudes were multiplied by a gain, such that target distance corresponded to 10–20% of maximum voluntary contraction and then averaged over the previous 50 ms. At each 50-ms time bin, the amplitudes of the targeted muscle pair were mapped to the position of a cursor along the cardinal [horizontal (X) and vertical (Y)] axes, respectively, and the cursor position was determined by a vector sum of these two components [12]. Thus, only activating muscles in isolation would move the cursor along the cardinal axes. Participants trained on three muscle pairs for six sessions each over 2 weeks. We assigned participants pseudo-randomly into three groups: two groups trained isometrically (restraining the arm) for either 60 or 90 min per session (ISO_60_ and ISO_90_, respectively) and one group trained without restraining the arm (movement group) for 90 min per session (MVMT). Further details of the training, including specific muscle pairs, are described in our previous study [13].

**Fig. 1:**
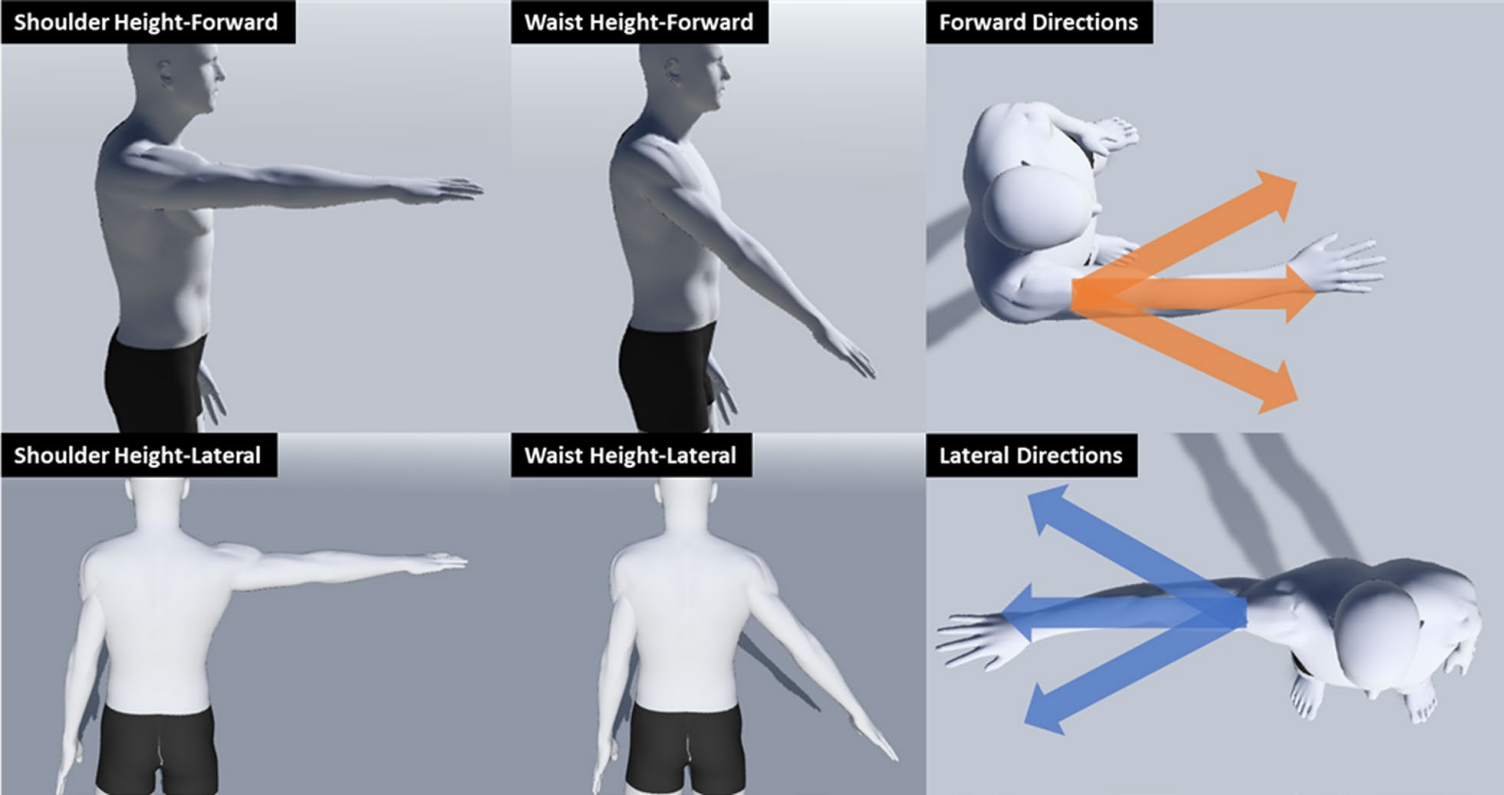
A reaching task that participants performed to assess the training effects using EMG data. During the motor assessment, participants performed reaches (only away-from-body reaches) three times to each of six targets (approximately 4″ in diameter and spaced 1ft apart), placed at the waist and shoulder height, in front of, and lateral to the impaired arm. The participants were asked to bring the arm back to the rest position at the side (elbow extended maximally, shoulder adducted, not flexed nor rotated with the thumb facing anteriorly) after each reach. The arrows indicate three directions of reach at the waist and shoulder height

To assess the effects of the training on motor impairment, an occupational therapist blinded to the training group evaluated FMA-UE at ﻿−2, 0, 2, 6, and 10 weeks relative to the start of training. The occupational therapist was not fully blinded to the number of weeks; they knew when the first evaluation was done, but we did not explicitly tell them the week number of the subsequent sessions. We recorded EMG while each participant performed a reaching task on weeks −2, 0, 2, 4, 6, and 10, relative to the training start. As depicted in Fig. 1, the task consisted of three reaches to each of six targets (approximately 4″ in diameter and spaced 1 ft apart), placed at waist and shoulder height, in front of, and then lateral to, the impaired arm (36 reaches, in total, to sample a broad range of reaching directions). The participants were asked to reach to the target as best as they could (self-paced speed) and no adjustments were made for severely impaired survivors. After each reach, participants were asked to bring the arm back to the rest position at the side (elbow extended maximally, shoulder adducted, not flexed nor rotated with the thumb facing anteriorly). The resting period in between each reach was at least 3 s.

### EMG recordings

EMGs were recorded to identify muscle targets for the MyoCI training and assess the effects of the training on intermuscular coordination in the arm after stroke. We identified co-activating muscles as targets for training in the study. We defined co-activation as the pairwise correlation coefficient between each pair of EMG envelopes during reaching. Muscle pairs with the largest abnormal co-activation—i.e., not seen in healthy arms during reaching—were selected for the MyoCI training. The selected muscle pairs typically included biceps/anterior deltoid, anterior/posterior deltoid, and triceps lateral head/posterior deltoid [13].

The skin was prepared by lightly exfoliating and cleaning with alcohol to collect EMG signals. We placed wireless, active EMG electrodes (Trigno, Delsys, Inc.) on the skin over the bellies of 8 muscles on the affected arm: anterior, middle, and posterior deltoids (AD, MD, and PD); biceps brachii (BI), triceps [long (TRIlong) and lateral (TRIlat) heads]; brachioradialis (BRD); and pectoralis major (PECT). EMG electrodes were placed according to guidelines of the Surface Electromyography for the Non-Invasive Assessment of Muscles—European Community project (seniam.org); a skin marker was used to ensure consistent EMG placement across sessions. EMG was sampled at 1926 Hz.

### Participants classification

The 32 participants, who completed both 6 weeks of training and the 10-week evaluation, were divided into two subgroups, responders and non-responders, based on their FMA-UE change after 6 weeks of training. Compared to the mean of the FMA-UE measured at weeks −2 and 0, if the score at week 6 increased by more than 2 points, the participant was considered a responder. For all analyses, we combined subjects across training groups (ISO_60_, ISO_90_, and MVMT).

### Data pre-processing and synergy analysis

Before the synergy identification, the raw EMG signals, recorded at weeks −2, 0, 2, 4, 6, and 10, were high-pass filtered at 50 Hz, rectified, then low-pass filtered (4th order Butterworth) at 5 Hz to obtain the envelope of the signals [14]. The processed EMG envelope of each muscle was normalized by dividing it by the maximum amplitude over all trials for each week to avoid any bias in synergy extraction toward the muscles with high-amplitude activation. For each subject, the normalized EMG envelopes, which included the entire segment from rest to target and back to rest, were then concatenated across all trials per week to identify one representative set of muscle synergies that counted the entire set of 36 reaches. A Kolmogorov–Smirnov test was used to test the normality of the data distribution, and the data were indeed normal (alpha = 0.05).

To identify the muscle synergies, we applied a non-negative matrix factorization (NMF) to the pre-processed EMG. In the NMF algorithm, the EMG was modeled as a synergy matrix (W) multiplied by the corresponding activation profile matrix (C):

1 $${EMG}_{reconstructed}=W\bullet C$$

The matrix *W* consisted of *N* column vectors, each of which described a muscle synergy with eight muscle weights. *C* was an *N* by *T* matrix, denoting activation of *N* synergies across the trials with *T* data samples. To estimate the optimal number of synergies that sufficiently reconstructed the spatial characteristics of the EMGs for each participant at each week, the minimum number of synergies that guaranteed the following criteria was identified: the global variance accounted for (gVAF) of the entire dataset greater than 90%, with less than a 5% increase in mean gVAF upon addition of another synergy (diffVAF) [7, 15]. The VAF value was defined based on the ratio between the summation of the squared errors (SSE) and the total sum of the squared with uncentered EMG (SST):

2 $$VAF=100\times \left(1-\frac{SSE}{SST}\right)$$

For quantifying the changes in the overall composition of muscle synergies between pre- and post-training of each participant, we compared norm synergies (the mean across participants) at the baseline week and week 6 using a similarity index (r-value; between 0 and 1) calculated from the scalar product [16]. The EMG of week 0 was typically used as the baseline EMG, while that of week −2 was alternatively used for seven subjects to ensure the quality of baseline EMG for further analysis.

To determine the significance of the similarity, we generated 1000 sets of 1 by 8 random synergy vectors consisting of randomly selected muscle weights of synergies identified in the study, and we performed the dot product of all possible pairs of random synergies [7, 17]. If the similarity between two muscle synergies exceeded the 95th percentile of the similarity indexes of random synergies (similarity threshold = 0.86), we considered the two muscle synergies statistically similar. We used the same method to quantify the inter-subject variability of synergy composition within a group. The norm synergy at each week was compared with its corresponding synergy of each participant using the dot product.

### Disparity index (DI) analysis

To explore the training effects on the synergies, the relative change in the muscle activation weights (*W*) of trained muscle pairs within a synergy between the baseline week and week 6, post-training, was computed. The synergies were extracted from EMG envelopes concatenated across all trials for each week, and each trial included the entire reaching segment from rest to target and back to rest. Therefore, the weights represent the activation patterns of arm muscles that can be combined to characterize the inter-muscular coordination during the entire set of 36 reaches. At the baseline week, we selected the trained muscle pair whose muscle weights (m1_base_ and m2_base_, for the first and second muscles of the pair, respectively) within a synergy exceeded a co-activation threshold level (${\text{Th}}_{{{\text{CoA}}}} = \frac{1}{\sqrt 8 } = 0.35$). Th_CoA_ was determined based on the normalized weight of each muscle within a synergy if all eight muscles were equally activated. Using the selected m1_base_ and m2_base_, we quantified the disparity index (DI) of a muscle pair at the baseline (DI_base_) as:

3 $$DI=\sqrt{{(m1-\frac{m1+m2}{2})}^{2}+{(m2-\frac{m1+m2}{2})}^{2}}$$

**Fig. 2:**
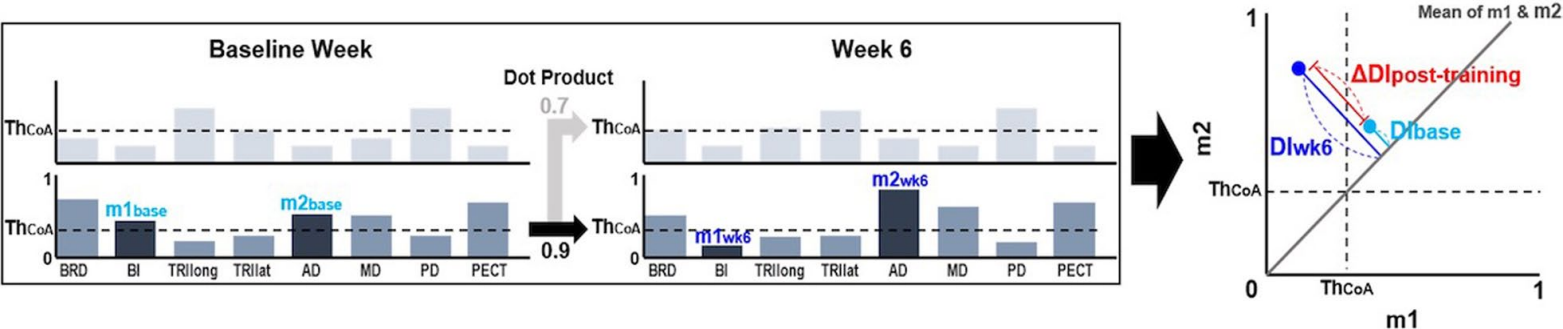
Calculating the disparity index (DI). From the synergy set of the baseline week, the synergy with the highest co-activation (lowest difference) between the targeted muscle pair (e.g., BI and AD in the second synergy (dark gray); m1_base_ and m2_base_, their muscle weights) is identified. Both m1_base_ and m2_base_ are higher than Th_CoA_, the threshold of muscle co-activation. The synergy at week 6 (the second synergy of week 6) corresponding to the synergy of the baseline week is selected by calculating the scalar product with the baseline synergy 2. DI is calculated (*right*) from m1 and m2 at both time points, as the distance from the mean of m1 and m2 (Eq. 3). Finally, the change in the muscle weight disparity after training (ΔDI_post-training_ in red) is obtained by subtracting DI_base_ from DI_wk6_

A DI value represents how much the pair of muscle weights diverged from their co-activation level, i.e., the mean of their weights. Therefore, a higher DI value indicated a lower level of co-activation of the muscle pair. After identifying m1_base_ and m2_base_ within a synergy and calculating DI_base_, we obtained the weights of the same muscles in a corresponding synergy at week 6 (m1_wk6_ and m2_wk6_) to calculate their disparity index (DI_wk6_). The selection of the corresponding synergy vector at week 6 was based on the similarity score calculated from the scalar product. Once DI_base_ and DI_wk6_ of the trained muscle pair were obtained, we calculated the change in the weight disparity (ΔDI_post-training_ = DI_wk6_—DI_base_) to quantify the training effect on the co-activation of the trained muscle pair (Fig. 2). If more than one synergy at the baseline week contained both m1_base_ and m2_base_ exceeding Th_CoA_, the synergy with the smallest weight disparity between the two muscles was considered the representative synergy for the co-activation of the given muscle pair. That is, we chose the synergy with the most co-activation between the muscle pair because that was what we aimed to counteract with the MyoCI training. To summarize, as depicted in Fig. 2, the algorithm selects the representative weights of each trained muscle pair from the baseline week (pre-training), which exceed the co-activation threshold and have minimal disparity, and compares them with the corresponding weights obtained from week 6 (post-training) using the DI method.

We measured the significance of the change in DI after six weeks of training in three different ways. First, we compared ΔDI_post-training_ with the 95% confidence interval of baseline variation, which we computed using 1000 bootstrap samples of the pre-training ΔDI values obtained from the entire participants at week 0 and week −2 (ΔDI_pre-training_ = DI_wk0_ − DI_wk-2_). To validate whether the Th_CoA_ level influenced the results, we compared ΔDI_post-training_ with the bootstrapped ΔDI_pre-training_ calculated using twenty different thresholds set at 5–100% of Th_CoA_ (in increments of 5%). Second, we computed the ΔDI_post-training_ for the trained muscle pair and for all possible pairwise combinations of untrained muscles. Third, we computed the significance of the difference in the distributions of DI_base_ and DI_wk6_ of all trained muscle pairs regardless of their initial weights (no Th_CoA_ applied) using a two-tailed t-test (alpha = 0.05).

## Results

### Estimation of the number of synergies and overall synergy composition

**Fig. 3:**
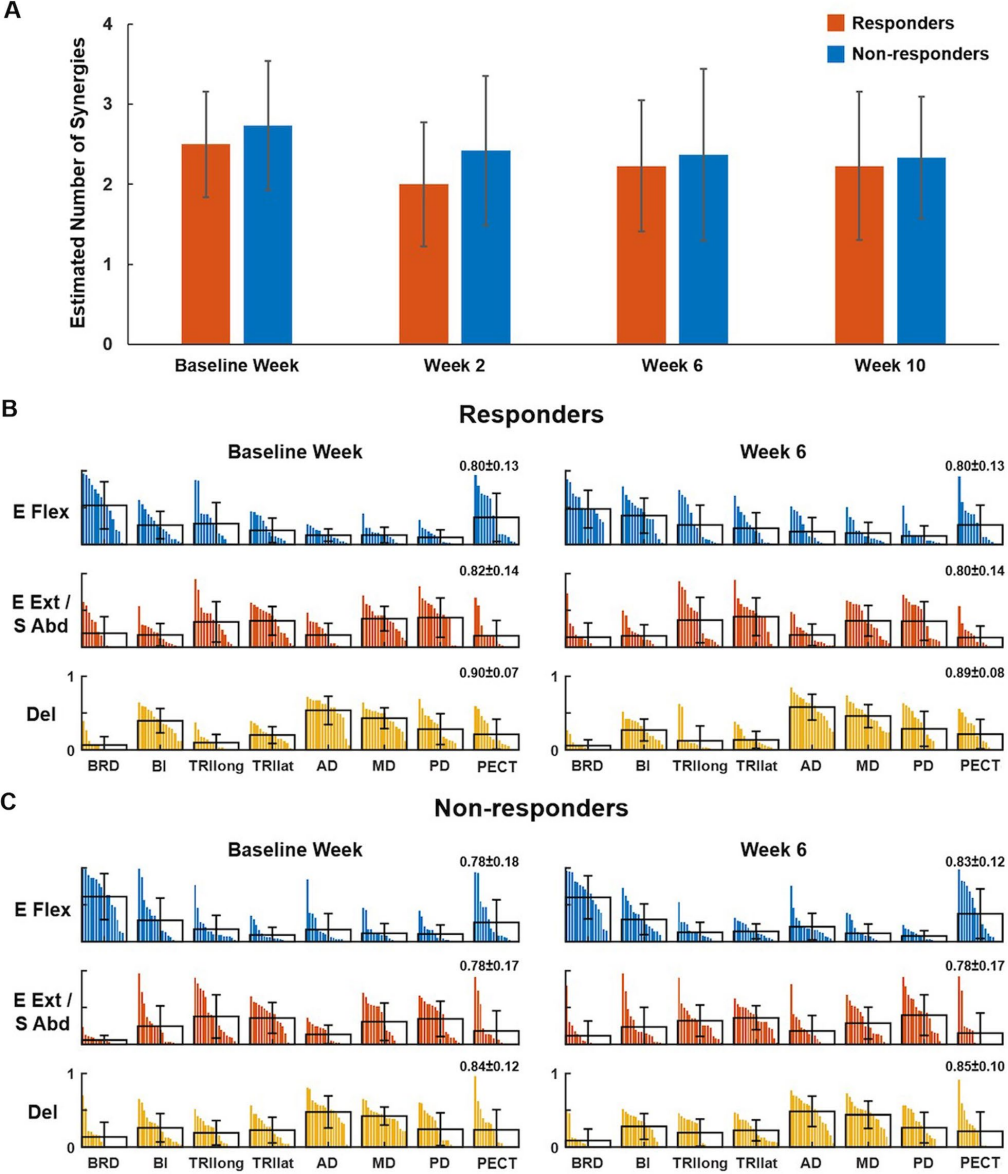
The number and composition of muscle synergies in responders and non-responders. **A** Estimation of the number of synergies identified across weeks including pre-training (Baseline week), interim (week 2), post-training (week 6), and retention (week 10) in the responder and non-responder groups. The number of synergies did not differ between the two groups nor across weeks in each group. **B, C**, The mean and SD of muscle weights, superimposed on the distribution of the muscle weights of responders (**B**, n = 15) and non-responders (**C**, n = 17), per each of three synergies identified in pre- and post-training (Baseline week and week 6, respectively). The muscle weights per muscle were displayed in descending order. Eight muscles included: brachioradialis (BRD); biceps brachii (BI); triceps (long (TRIlong) and lateral (TRIlat) heads); anterior, middle, and posterior deltoids (AD, MD, and PD); and pectoralis clavicular fiber (PECT). The synergies were characterized as (1) E Flex: elbow flexors, (2) E Ext/S Abd: elbow extensors, and 3) Del: three heads of the deltoid. The r-value next to each muscle synergy indicates the scalar products between the norm synergy (the mean across the subjects) and its corresponding synergy of each participant (mean ± SD)

Based on the level of improvement in the motor impairment (FMA-UE) after the training, 15 and 17 participants were categorized as responders and non-responders, respectively. Typically, two to four synergies were identified from the EMGs of each participant before, interim, and after the MyoCI training in both responder and non-responder groups (Fig. 3A). The average number of synergies across all participants and weeks was 2.41, indicating that three synergies predict most of the total EMG variance in both groups and across weeks. Overall, no consistent pattern of change in the number of muscle synergies was observed after the training in each group. The number of synergies did not change significantly from the baseline to week 6 (change in responders (n = 15): − 0.27, *p* = 0.37; non-responders (n = 17): − 0.37, *p* = 0.19, one-way ANOVA). Compared to the baseline week, the number of synergies was preserved in 40.6% (n = 13) of all participants, decreased in 37.5% (n = 12), and increased in 21.9% (n = 7). Among responders, the number of synergies was preserved in 46.7% (n = 7), reduced in 33.3% (n = 5), and increased in 20.0% (n = 3). Among non-responders, 35.3% (n = 6) showed no change in synergy number, 41.2% (n = 7) decreased, and 23.5% (n = 4) increased.

The composition of three muscle synergies extracted from each participant at each week was distinctive with the dominant muscle weights but with inter-subject variability. The synergies consisted of (1) two elbow flexors (BRD and BI) with PECT, (2) two elbow extensors (TRIlong and TRIlat) and shoulder abductor/extensor (MD and PD), and (3) three heads of the deltoid (AD, MD, and PD) with BI for both responders and non-responders (Fig. 3B and C, respectively). On average, the similarity (r-value) of three norm synergies (the mean of clustered synergies; Fig. 3B and C) between pre- (baseline week) and post-training (week 6) was 0.98 ± 0.01 and 0.99 ± 0.01 for responders and non-responders, respectively. The high similarity values indicated no significant change [i.e., r-value > the similarity threshold (0.86); *p* < 0.05] in the group-wise overall composition of muscle synergies due to the MyoCI training. However, when the norm synergy per group and week was compared with its corresponding synergy of each participant, inter-subject variability of synergy composition within the same group was observed (Figs. 3B and C). The mean r-value of each synergy was lower than the similarity threshold (0.86; *p* < 0.05), indicating substantial inter-subject variability of the synergy composition [responders: 0.84 ± 0.11 (baseline week), 0.83 ± 0.12 (week 6); non-responders: 0.80 ± 0.15 (baseline week), 0.82 ± 0.13 (week 6)]. Also, the same result was obtained even when extracting only two, instead of three, synergies (*p* < 0.05). In terms of the activation profile, regardless of the number of synergies extracted (two or three), there was no clear pattern of change in the mean magnitude of coefficients observed in both groups (p > 0.05). Overall, the observations motivated us to develop a more precise way to quantify potential changes in a pair of muscle weights within a synergy, trained in the MyoCI protocol.

### Weights of the trained muscle pairs within a synergy changed with MyoCI training

**Fig. 4:**
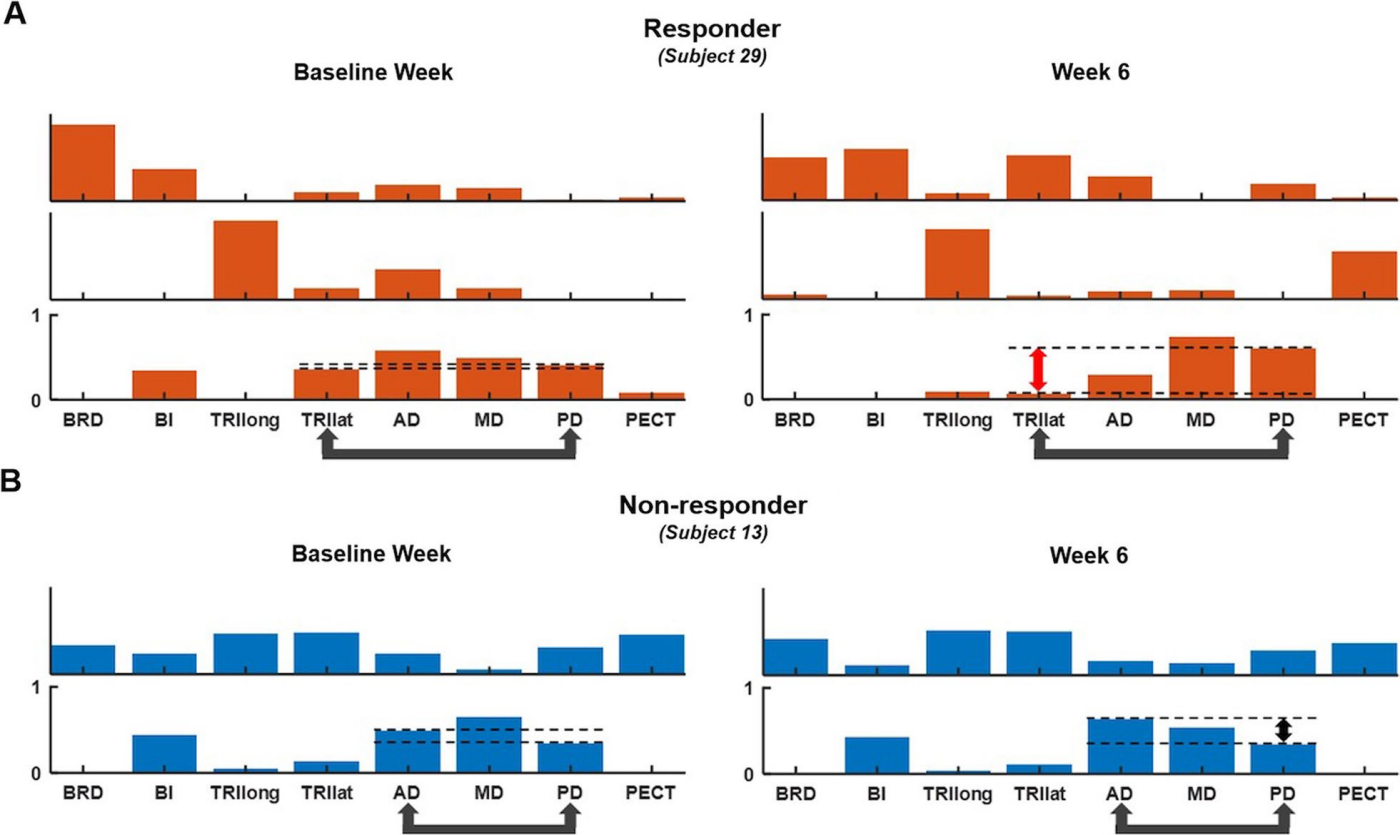
Exemplary synergies identified pre- and post-training (Baseline week and week 6) of a responder (**A** Subject 29) and a non-responder (**B** Subject 13). Arrows below the abbreviation of muscle names indicate the first trained muscle pair for each participant. Dashed lines show the difference between the weights of the trained muscle pair

The MyoCI training modulated the weights of trained muscle pairs within a synergy in responders, not in non-responders. Figure 4A shows an example of these patterns for a representative responder; the relative weights of the first trained muscle pair, the TRIlat and PD, in the synergy noticeably changed after the training. This finding signifies a reduction in co-activation of the first muscle pair. The decrease in co-activation was not typically observed in non-responders (Fig. 4B). These results suggest that the MyoCI training, which aimed to decrease the abnormal co-activation of a pair of muscles, increased the disparity of weights of the trained muscle pair within a given synergy in responders but not in non-responders.

**Fig. 5:**
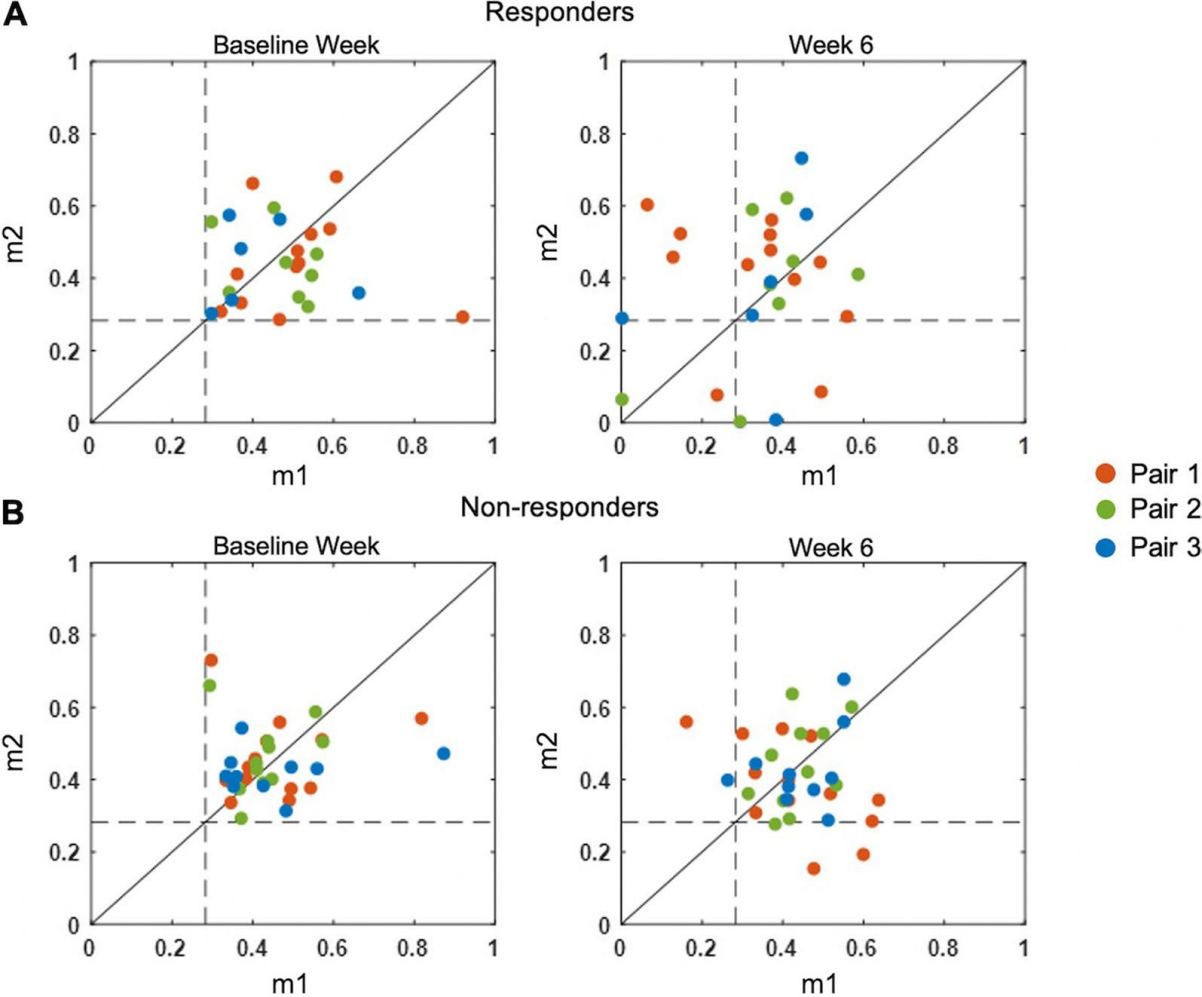
The two-dimensional mapping of activation weights of the trained muscle pairs whose initial co-activation level exceeded Th_CoA_ (dashed lines). m1 and m2 indicate the activation weights of a pair of trained muscles, muscles 1 and 2. Red, green, and blue indicate the muscle pairs trained for the first, second, and third two weeks in the MyoCI training, respectively. The disparity index (DI) measured the average of the distance between each dot and the solid diagonal line (representing co-activation since the weights were equal) for responders (**A**) and non-responders (**B**). The increase in DI indicated a decrease in co-activation

Before MyoCI training (baseline), the mean DI, computed with Th_CoA_ applied, of all three targeted muscle pairs was comparable between responders and non-responders (Fig. 5; DI_R-base_ = 0.091 ± 0.095, DI_NR-base_ = 0.074 ± 0.076; *p* = 0.33, t-test). After the training (week 6), only the responders showed an increase in DI compared to baseline (**Δ**DI_R_ = 0.044 ± 0.11, *p* = 0.046; **Δ**DI_NR_ = 0.018 ± 0.11, *p* = 0.10; t-test), which implied a decrease in co-activation of the trained muscle pair within the synergy in general. Interestingly, in both groups of participants, the first trained muscle pair, which initially had the highest abnormal co-activation (pairwise correlation) among the three pairs, demonstrated the most reduction in co-activation due to MyoCI training (**Δ**DI_R-pair1_ = 0.073 ± 0.13, **Δ**DI_NR-pair1_ = 0.055 ± 0.14; **Δ**DI_R-pair2_ = 0.002 ± 0.055, **Δ**DI_NR-pair2_ = 0.011 ± 0.092; **Δ**DI_R-pair3_ = 0.042 ± 0.14, **Δ**DI_NR-pair3_ = − 0.021 ± 0.065; Fig. 5). However, only the responders showed a significant reduction of DI in the first pair (*p* = 0.016, t-test).

**Fig. 6:**
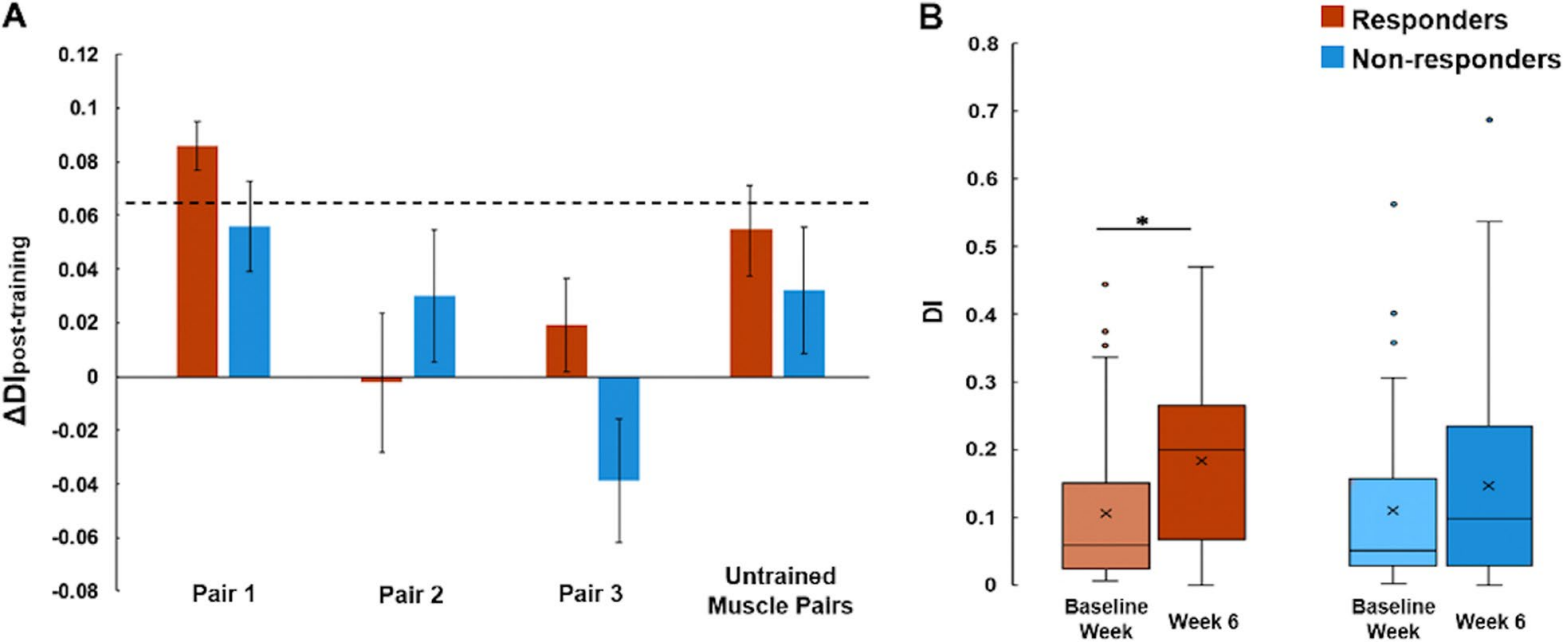
Changes in the disparity of activation weight of the trained muscle pairs. **A** ΔDI_post-training_ (mean ± SD) of each of three trained muscle pairs and all possible pair-wise combinations of untrained muscles in responder and non-responder groups when 5–100% of the Th_CoA_ was applied. The dashed line indicates the average 95th percentile of the bootstrapped ΔDI_pre-training_. **B** DI of all of the first trained muscle pairs, computed pre- and post-training in the responder and non-responder groups, respectively, when no weight threshold was applied (*p = 0.011)

This result was not influenced by the level of Th_CoA_ because ΔDI_post-training_ was comparably similar while the threshold was varied systematically (5–100% of Th_CoA_ with an increment of 5%). Among the three pairs of muscles trained, only **Δ**DI_R-pair1_ in responders exceeded the 95^th^ percentile level of ΔDI_pre-training_ regardless of the level of Th_CoA_ (*p* = 0.0025; Fig. 6A). In particular, the participants in the movement-based training group showed a greater ΔDI_post-training_ value of the first trained muscle pair than the isometric training groups (*p* = 0.0029 for comparing MVMT to ISO_60_; *p* < 0.001 for comparing MVMT to ISO_90_, ANOVA). For non-responders, the ΔDI of the first pair did not significantly change (*p* = 0.12). Similar results were observed when there was no threshold, Th_CoA_, applied. These findings mean that, even when the DIs of all of the first trained muscle pairs across participants were considered regardless of their level of initial co-activation, the responder group demonstrated a statistically significant increase in the mean DI at week 6 (*p* = 0.011; Fig. 6B).

## Discussion

In this study, we investigated the effect of MyoCI training on intermuscular coordination using muscle synergy analysis. The number of synergies identified during reaching ranged from two to four and did not change due to the training. The overall composition of muscle synergies also did not change. However, there was a notable change, within individual synergies, in the disparity of muscle weights of the trained muscles in responders to the training. While not all muscle pairs changed their co-activation, the muscle pairs with the largest abnormal co-activation before training showed the largest reduction in coupling after training, especially in the movement-based training group. These results suggest that our MyoCI paradigm did, in fact, allow participants to decouple the targeted muscle pairs (particularly the most abnormally co-activating, muscle pair 1), which, in turn, was correlated with improved movement. The fact that the training only changed the behavior of the trained muscles suggests that the CNS is capable of making highly fractionated changes in motor control, even after a stroke-causing severe impairment.

Our prior study showed that MyoCI training might reduce arm impairment in moderately and severely impaired stroke survivors [13]. That study found some reduction in pairwise correlations of the trained muscles during reaching in the movement group, but not in the isometric groups. This study, which looked at the within-synergy weights of the trained muscles, found increased disparity (reduced co-activation). Thus, the more comprehensive approach to intermuscular coordination using muscle synergy analysis revealed changes that were not seen in the simpler, pairwise correlation analysis.

In this study, we found no consistent pattern of change (e.g., merging or fractionation) observed in the overall composition of the synergy sets. Few studies have characterized the number of muscle synergies after stroke, as compared to unimpaired limbs, as having three different patterns: preservation, merging, and fractionation of unimpaired synergies [9, 18, 19]. More severe impairment after stroke correlated with having fewer synergies (merging) [18, 20] or fewer muscle networks [21]. This observation suggests that we might expect to see fractionation of synergies (greater number of synergies) with improved function from MyoCI training. However, the current MyoCI paradigm was customized for each individual’s pattern of muscle co-activation, meaning that different muscle pairs were targeted for different participants. This inter-subject variability may have resulted in a diverse effect on the synergy composition across participants, given the variation in initial co-activation of muscles trained. Moreover, no clear pattern of change in the activation profile was observed due to the inconsistency of the pattern of change in the number and the composition of muscle synergies that arose from the high inter-subject variability.

To account for this diversity, we designed DI analysis to focus on the training effect on each individual’s muscle synergies, particularly those most relevant to the targeted muscle pairs. Previous studies showed that conventional synergy analysis can be misleading [22, 23]. When synergies are identified from a small subset of muscles, the conventional method using the VAF and its preset threshold for synergy identification can overestimate VAF, which affects the estimation of the number of synergies and the corresponding synergy composition [22]. This overestimation can lead to ignoring important features embedded in the residual muscle activities [23]. DI analysis captured a consistent change in synergy activation weights in the responders that was not identified by conventional synergy analysis. DI thus complements conventional synergy analysis. However, the current DI method is limited to reflecting the change in the disparity between weights of a muscle pair only.

Only a few recent studies have investigated the effects of rehabilitation training on potential changes in the number and/or composition of muscle synergies and/or their activation profiles in stroke survivors [11, 24, 25]. Two small studies showed that EMG feedback could reduce co-activation between agonist and antagonist muscles during training in stroke participants [26, 27]. While this finding aligns with our overall result, these studies did not assess the more global picture of arm intermuscular coordination. Pierella et al. [28] showed that improved arm function in the subacute stage, coinciding with exoskeleton training, correlated with an increase in muscle synergy number and similarity to non-stroke controls in some of the participants. Two other studies showed some evidence of post-stroke synergies becoming more like those of unimpaired arm movement due to different rehabilitation strategies [29, 30]. However, other studies showed unclear relationships between improved motor outcomes and either the number of muscle synergies or similarity between impaired and unimpaired synergies [31, 32]. Notably, these last two studies are pilot studies that included participants with only mild to moderate impairment.

One of the characteristics of human upper limb movements is that they can be highly fractionated—i.e., we can precisely control the activation of individual joints and even single muscles. This is thought to be due to the precise connections of the corticospinal tract to small pools of motoneurons [33]. The presence of abnormal co-activation after stroke means that less fractionated movements are possible; this is thought to be due to corticospinal tract damage and higher reliance on the reticulospinal tract after stroke [34–37]. Here, we showed that MyoCI training enabled stroke survivors to improve arm function not by changing entire synergies but rather by reweighting the activities of just those muscles that were targeted. This finding indicates that even severely impaired stroke survivors are capable of fractionated changes in movement control. This, in turn, suggests that some corticospinal tract functions may have remained in our responders to MyoCI. Alternatively, it is possible that plastic changes occurred at the spinal cord level. While we unfortunately did not directly examine the corticospinal excitability (e.g., with transcranial magnetic stimulation) or cortico-reticulospinal responses such as StartReact [36, 38], we hope to examine this in future studies to determine the extent to which this change is dependent on corticospinal tract changes. Regardless of the exact mechanism, MyoCI training enabled highly precise targeting of changes in muscular coordination.

This study did have some limitations. The muscle synergies were not investigated in the arms of neurologically intact persons or in the ipsilesional arm of stroke survivors. Therefore, the comparison of synergies was made only within the stroke-impaired arm. Moreover, since the total number of analyzed muscles affects synergy analysis [22], further testing is important to examine whether the results remain consistent when more than eight arm muscles are examined. We are testing this in a follow-up, sham-controlled study with a wearable version of the MyoCI [39]. Further, some muscles may co-activate normally at different times. Overall, we selected those muscle pairs for training that were typically not synergistic in multiple activities of daily living, in particular reaching, i.e., those that showed significantly less co-activation in age-matched, able-bodied individuals performing the same reaching task. Lastly, there is no widely accepted definition of “co-activation” in terms of the weight of muscle within a synergy, which makes it unclear how best to quantify the degree of co-activation. The proposed DI method focuses on the change in weight disparity of a muscle pair to quantify the change in pair-wise co-activation level. This approach simplifies the quantification of change in co-activation. However, other factors, such as the change in the mean weight of a muscle pair, could be also considered in future studies to interpret the training effect on the intermuscular coordination more comprehensively.

## Conclusions

In conclusion, we demonstrated that the MyoCI paradigm improved the upper extremity motor impairment of the stroke survivors by rebalancing the muscle weights of trained muscles within muscle synergies. While there was no consistent pattern of change in the number and composition of muscle synergies after MyoCI training, the DI analysis captured the increase in weight disparity of trained muscle pairs within a synergy in the responders. Although not every muscle pair was decoupled, the results imply that MyoCI training can target abnormal co-activation, particularly of the most abnormally co-activating muscle pair. Further, MyoCI can enable functional improvement in people with severe impairment from stroke, even years after the stroke occurs.

## Data Availability

The data used in this study may be made available by the corresponding author upon a reasonable request.

## Abbreviations

MyoCI: Myoelectric computer interface
DI: Disparity index
FMA-UE: Fugl-Meyer Assessment of the upper extremity
